# Supplementary material for: A pilot study of alternative substrates in the critically Ill subject using a ketogenic feed
Source: Nat Commun. 2023 Dec 15;14:8345. doi: 10.1038/s41467-023-42659-8 (PMC10724188; doi:10.1038/s41467-023-42659-8)
Supplement: Supplementary file 1 — Supplementary Information [file 41467_2023_42659_MOESM1_ESM.pdf]

Alternative Substrates in the Critically Ill Subject (ASICS): A Randomised Pilot Study to Assess the Safety, Feasibility, Tolerability and Metabolic Profiling of a Novel Ketogenic Feed

SUPPLEMENTARY INFORMATION

Angela McNelly[1], Anne Langan[2], Danielle E. Bear[3,4], Alexandria Page[5], Tim Martin[5], Fatima Seidu[5], Filipa Santos[5], Kieron Rooney[6], Kaifeng Liang[1], Simon J Heales[7], Tomas Baldwin[8], Isabelle Alldritt[9], Hannah Crossland[9], Philip J. Atherton[9], Daniel Wilkinson[9], Hugh Montgomery[10,11], John Prowle[1,5], Rupert Pearse[1,5], Simon Eaton[8] and Zudin A. Puthucheary[1,5*]

[1]William Harvey Research Institute, Faculty of Medicine & Dentistry, Queen Mary University of London, [2]Department of Dietetics, Adult Critical Care Unit, Royal London Hospital, London, [3]Department of Nutrition and Dietetics St Thomas' NHS Foundation Trust, London, [4]Department of Critical Care, Guy's and St. Thomas' NHS, London, [5]Adult Critical Care Unit, Royal London Hospital, London, [6]Department of Critical Care, Bristol Royal Infirmary, Bristol, [7]Genetic & Genomic Medicine Department, UCL Great Ormond Street Institute of Child Health, London, [8]Developmental Biology & Cancer, UCL Great Ormond Street Institute of Child Health, London, [9]Centre of Metabolism, Aging & Physiology (COMAP), MRC-Versus Arthritis Centre for Musculoskeletal Aging Research & NIHR Nottingham BRC, University of Nottingham, Nottingham, [10]University College London (UCL), [11]UCL Hospitals NHS Foundation Trust (UCLH), National Institute for Health Research (NIHR) Biomedical Research Centre (BRC), London.

***CORRESPONDENCE TO**
Dr Zudin Puthucheary
Critical Care and Perioperative Medicine Research Group,
Adult Critical Care Unit,
Royal London Hospital,
London, E1 1BB
United Kingdom
Email: z.puthucheary@qmul.ac.uk

Supplementary Results

Recruitment and Retention

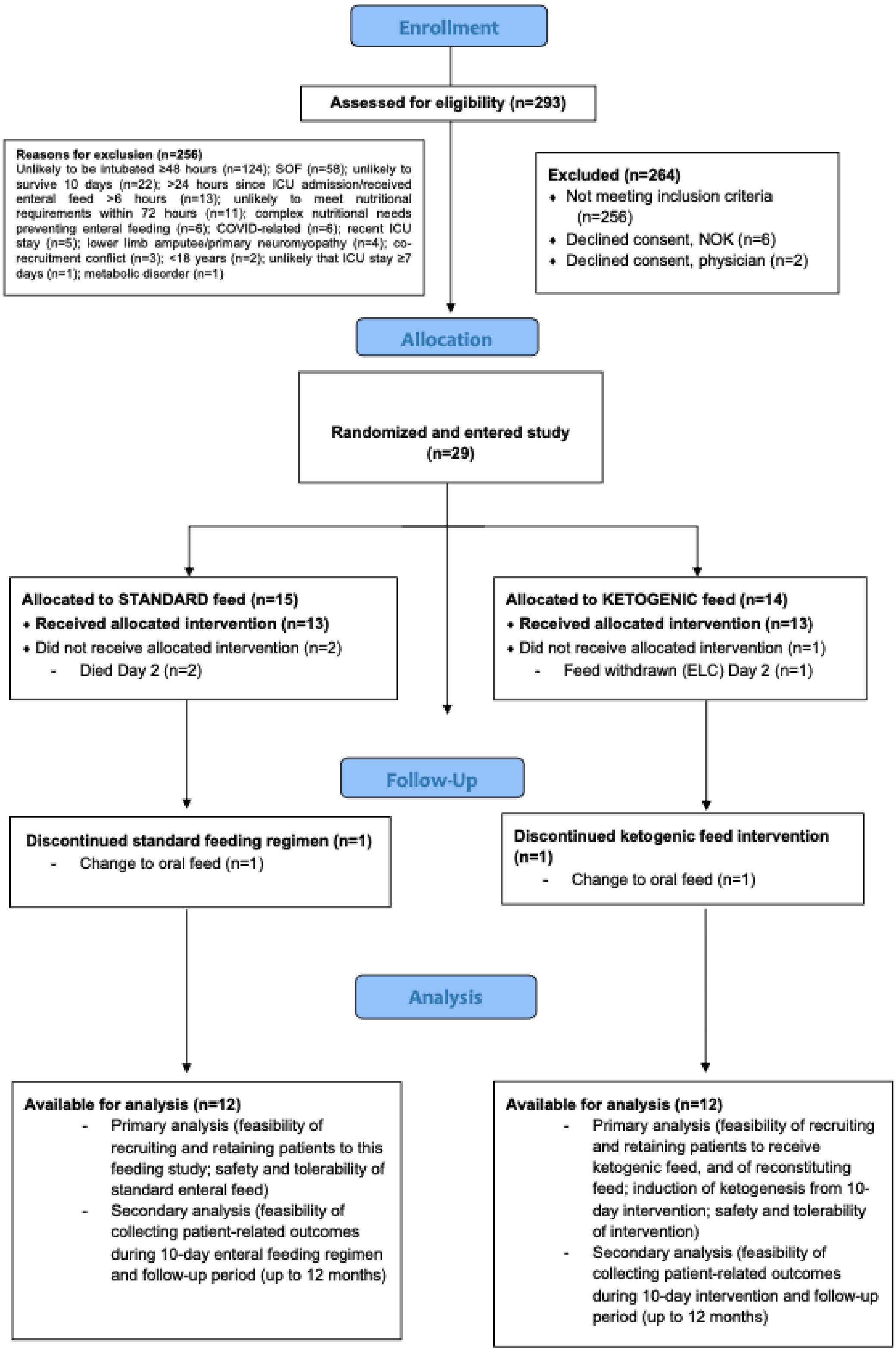

Figure S1: Consort flow diagram

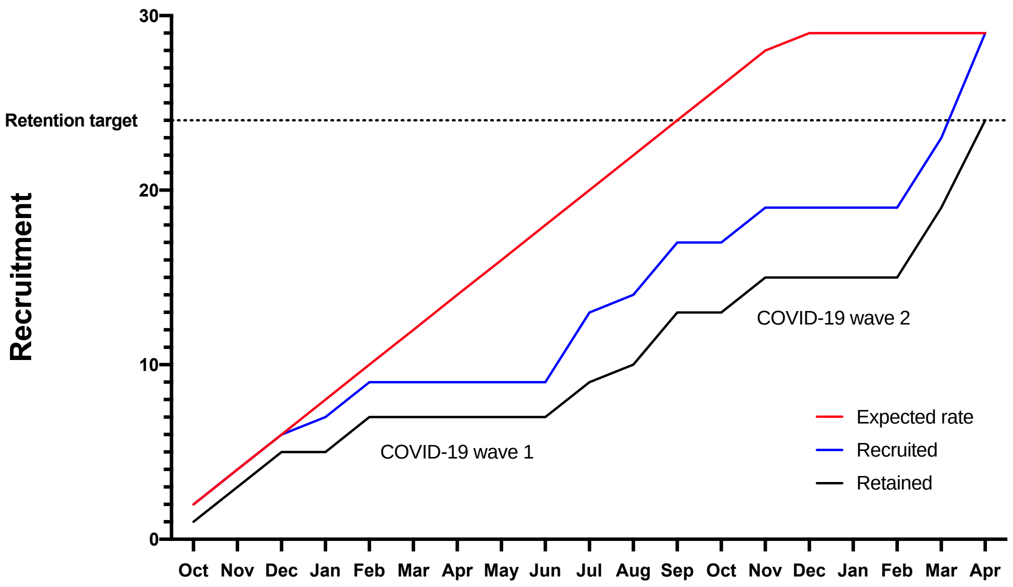

Figure S2: Recruitment rates and the effects of COVID-19 waves during the pandemic

Site	No. Screened	No. Recruited (%)	No. Retained (%)
**RLH**	258	27	23
**BRI**	35	2	1
**TOTAL**	293	29 (9.9)	24 (8.2)

Table S3: Recruitment across both sites. No.=number; RLH=Royal London Hospital BRI=Bristol Royal Infirmary

Withdrawal reason	Ketogenic Feed Arm (n=14) No. (%)	Standard Feed Arm (n=15) No. (%)
**ICU d/c<7d (died)**	0	2
**Feed withdrawn (ELC)**	1	0
**Feed change to oral**	1	1
**Total**	2 (14.3)	3 (20)

Table S4: Reasons given for premature withdrawal of participants. ICU= Intensive Care Unit; d/c<7d=discharged in less than 7 days; ELC=End of life care.

Acceptability and Feasibility

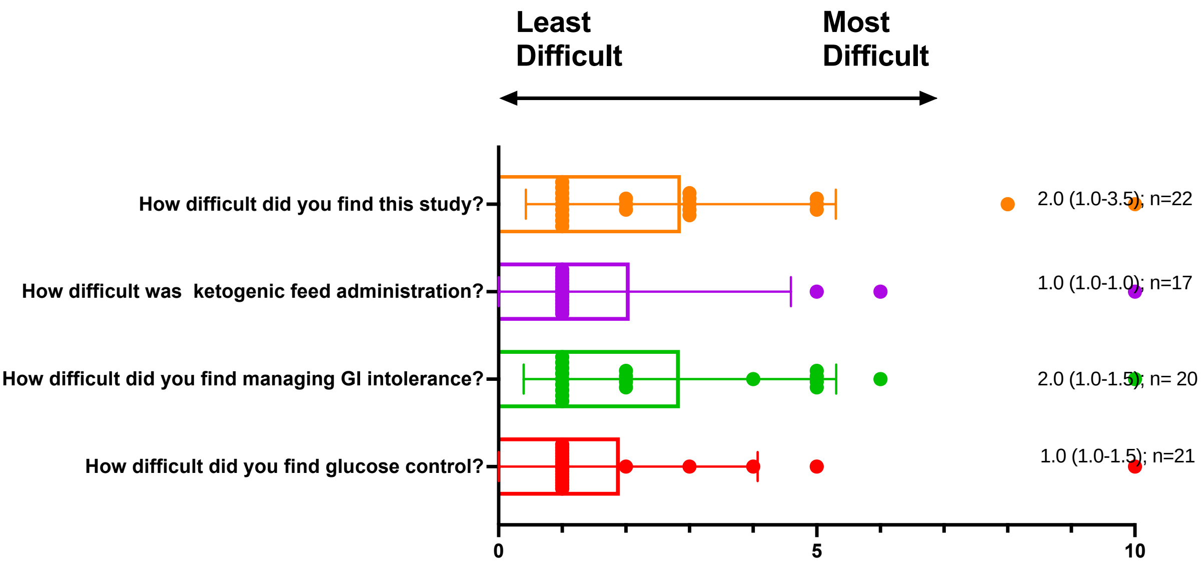

" Lack of pharmacists on study meant people were coming to me all day"
"4 Hour bags were inconvenient and increased work load, 24-hour bag was better"

Figure S3: Staff questionnaire data on feasibility and acceptability of delivering the intervention Data are median (Inter-Quartile Range) scores from n=23. Variation in data come from the multi-disciplinary sample population i.e. not all members of the team were involved in all aspects of the study

Safety data

Serious Adverse Event summary	Outcome
**Brain stem death due to trauma-related dissection of carotid arteries**	Unrelated
**Death from pulmonary embolism and biventricular failure**	Unrelated
**Metabolic acidemia secondary to hyperchloremia and dapaglifozine**	Unrelated; Clinicians stopped dapaglifozine and continued ketogenic feed
**Death from chest sepsis**	Unrelated

Table S5: Severe Adverse Events and trial related outcomes.

Days/events reported, No. (% total days/events)	High GRV*	Vomiting**	Diarrhoea (3-days)[a]	Diarrhoea (daily)[b]	Prokinetic Use	Hypoglycaemia^	Hyper-glycaemia^^
**STANDARD ENTERAL FEED (n=15)**	25(19.8)	7 (5.5)	8 (53.3)	54 (42.9)	29 (22.8)	2 (1.6)	73 (57.5)
**KETOGENIC ENTERAL FEED (n=14)**	19 (17.3)	9 (8.1)	10 (76.9)	54 (53.5)	23 (20.7)	0 (0.0)	29 (26.9)

Table S6: **Adverse events** *Days with Gastric Residual Volume ≥300mls; ^Blood glucose ≤3.9mM; **Days with any vomiting (>10mls); ^^Blood glucose ≥10mM; [a]Three-day episodes of Bristol Stool Score T6 or T7; [b]Days with Bristol Stool Score T6 or T7. GRV=Gastric Residual Volume.

	**Events reported, No. (%)**		**Mean (95% CI)**	
	AKI	Metabolic acidosis	Base Excess	Bicarbonate
**STANDARD ENTERAL FEED (n=15)**	1 (6.6)	0 (0.0)	1.78 (2.61-0.95)	26 (16.8-25.2)
**KETOGENIC ENTERAL FEED (n=14)**	1 (7.1)	2 (14.3)[#]	-1.68 (-1.01- -2.35)	22.6 (23.3-21.9)

Table S7: Adverse events #Deemed by clinical team to be related to the ketogenic feed. AKI=Acute Kidney Injury.

Nutritional Medium Chain Triglyceride concentrations and micronutrient delivered

Day	**D1**	**D2**	**D3**	**D4**	**D5**	**D6**	**D7**	**D8**	**D9**	**D10**
Mean (%) MCT	**44.4**	**53.6**	**62.1**	**69.1**	**77.1**	**76.3**	**76.1**	**74.5**	**73.9**	**74.0**
upper level	52.8	61.9	69.9	78.4	86.1	86.4	88.1	87.3	84.3	83.8
lower level	36.0	45.4	54.2	59.9	68.1	66.1	64.1	61.7	63.5	64.2

Table S8: Concentrations of Medium Chain Triglycerides delivered per day as part of the ketogenic feed. Data are mean % (95%CI) of estimated energy requirements.

Micronutrient	**Ketogenic Arm**	**Control Arm**
Vitamin A (μgRE)	9060	9379
Vitamin D (μgRE)	50	124
Vitamin E (mg)	110	147
Vitamin C (mg)	600	825
Vitamin B1 (mg)	14	14
Vitamin B2 (mg NE)	16	17
Niacin (mg)	180	324
Vitamin B6 (mg)	20	17
Folic Acid (μg)	2000	2616
Vitamin B12 (μg)	13	29
Biotin (μg)	1500	486
Pantothenic Acid (mg)	60	52
Vitamin K (μg)	200	644
Calcium (mg)	2000	7907
Phosphorus (mg)	1450	5849
Iron (mg)	140	132
Magnesium(mg)	1000	2190
Zinc (mg)	150	115

Iodine (μg)	1500	1337
Copper (μg)	10000	12900
Manganese (mg)	10	27
Chromium (μg)	250	657
Selenium (μg)	550	655

Table S9 Mean total micronutrient delivery over the 10 day intervention period

Urinary Ketone Body Concentration

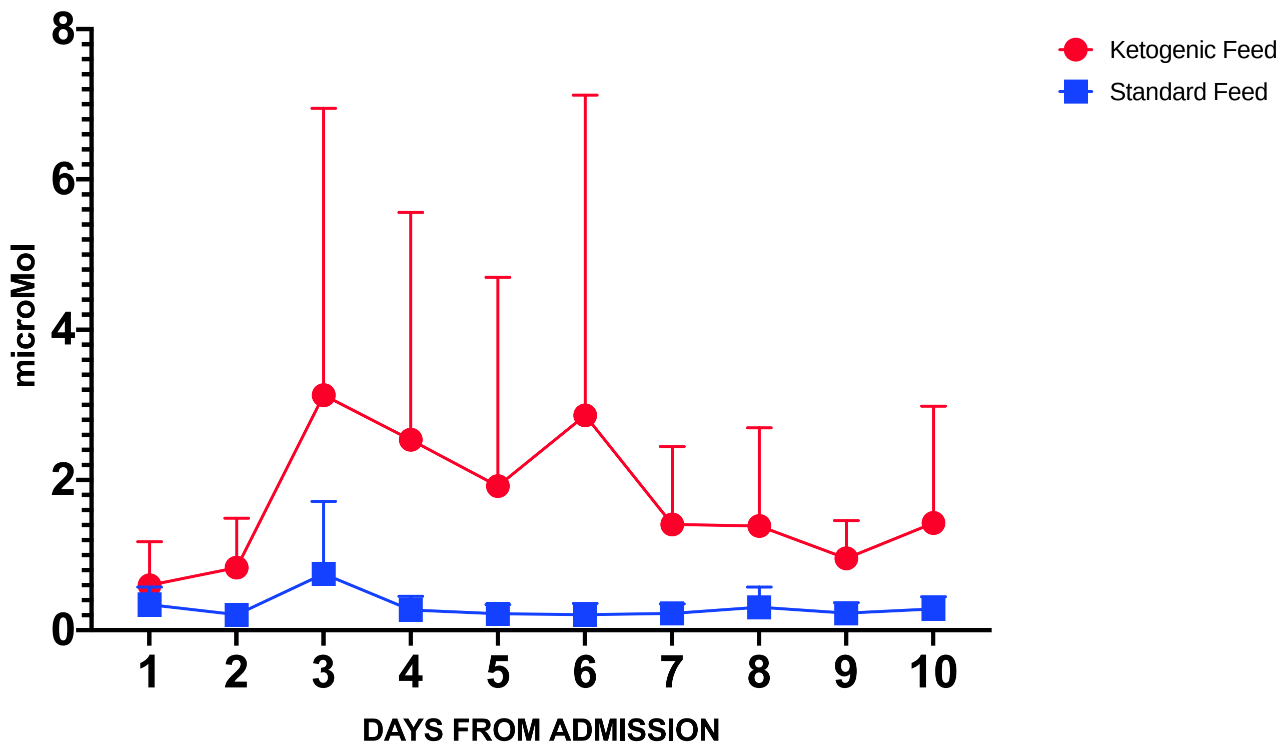

Figure S4: Urinary beta-hydroxybutryrate during the 10-day intervention. Red lines represent ketogenic feeding, and blue lines controls. N=14 subjects in the ketogenic arm and n=15 subjects in the control arm

Plasma Fatty Acid Concentrations

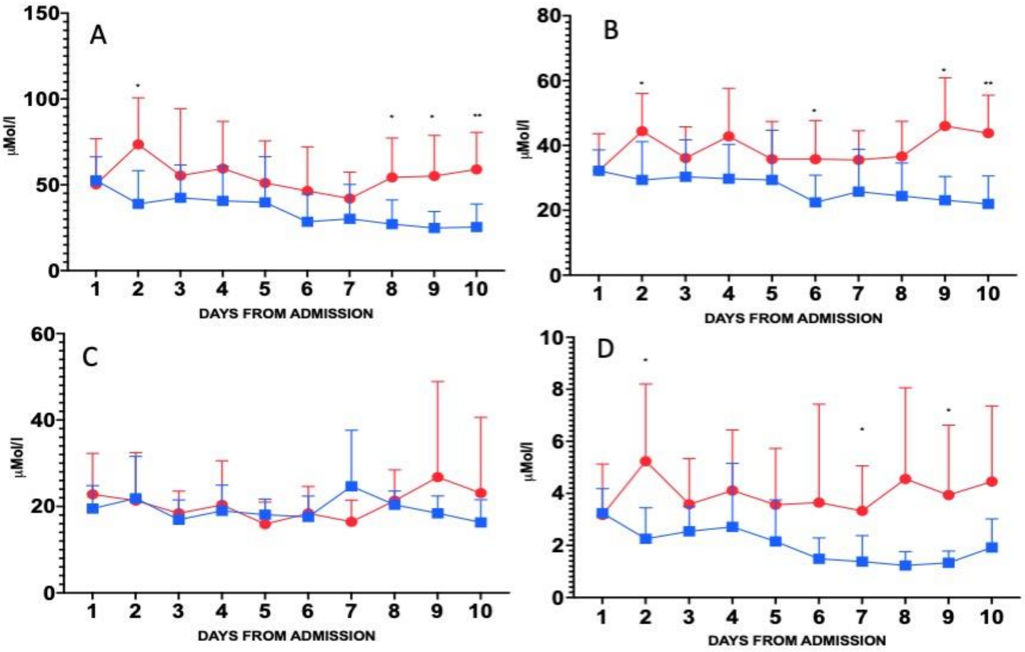

Figure S5: Octanoic acid (A), decanoic acid (B), and dodecanoic acid (C) in plasma during the 10-day intervention; octanoic:dodecanoic acid ratio (D). *p<0.05, **p<0.01 between arms (Two-tailed Mann Whitney-U test). N=14 subjects in the ketogenic arm and n=15 subjects in the control arm

Glycaemic analysis.

The AUC of plasma glucose was higher in the control arm than in the intervention arm (367.8 vs. 316.9).

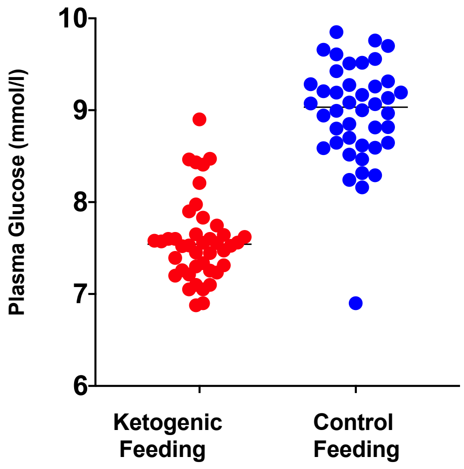

Figure s6: Raw plasma glucose data across both trial arms.102 glucose readings in ketogenic arm vs 128 glucose readings in the control arm

Respiratory Quotients

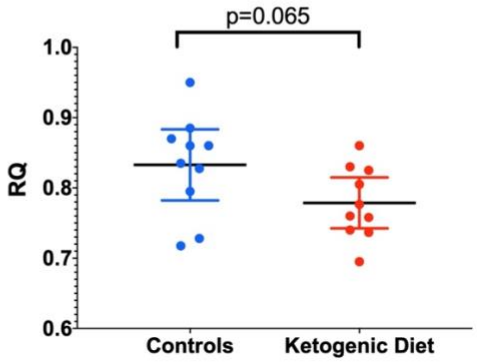

Figure S7: Respiratory Quotient (RQ) of a sub-group of patients receiving the ketogenic (red) and control (blue) feeds. n=9 controls, n=9 ketogenic feeding

Metabophenotyping

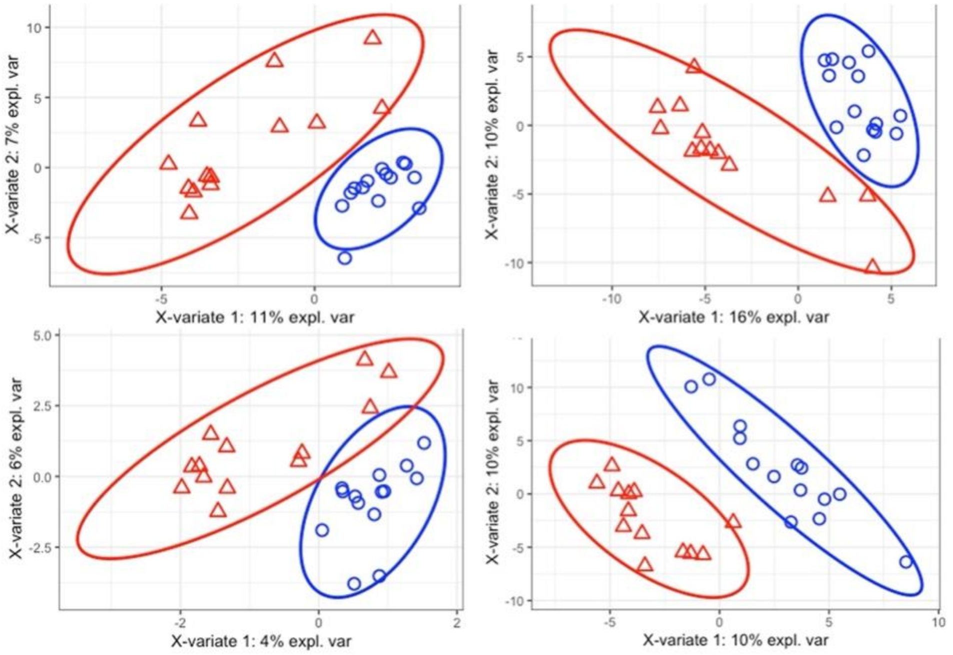

Figure S8: Sparse Partial Least Squares Discriminant analysis of patient randomised to ketogenic feeding on Day 1 (red triangle) and control feeding on day 1 (blue sphere). Clockwise from top right: polar positive, non-polar positive, non-polar negative, polar negative. Error rates are >20% (17%, 41%, 31% and 20% respectively), suggesting plot is overfitted and not true variance. N=14 subjects in the ketogenic arm and n=15 subjects in the control arm

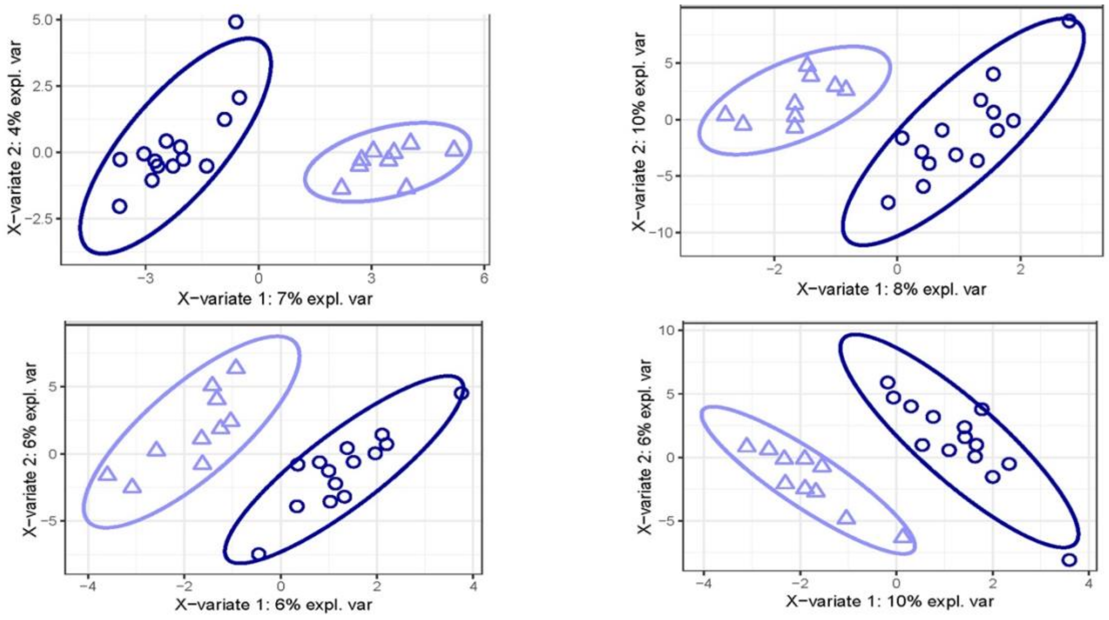

Figure S9: Sparse Partial Least Squares Discriminant analysis of patient randomised control feeding on Day 1 (blue sphere) and day 10 (blue triangle). Clockwise from top right: polar positive, non-polar positive, non-polar negative, polar negative. Error rates are mixed >20% (17%, 25%, 15% and 25% respectively), suggesting plot is at risk of overfitting a opposed to true variance. N=14 subjects in the ketogenic arm and n=15 subjects in the control arm

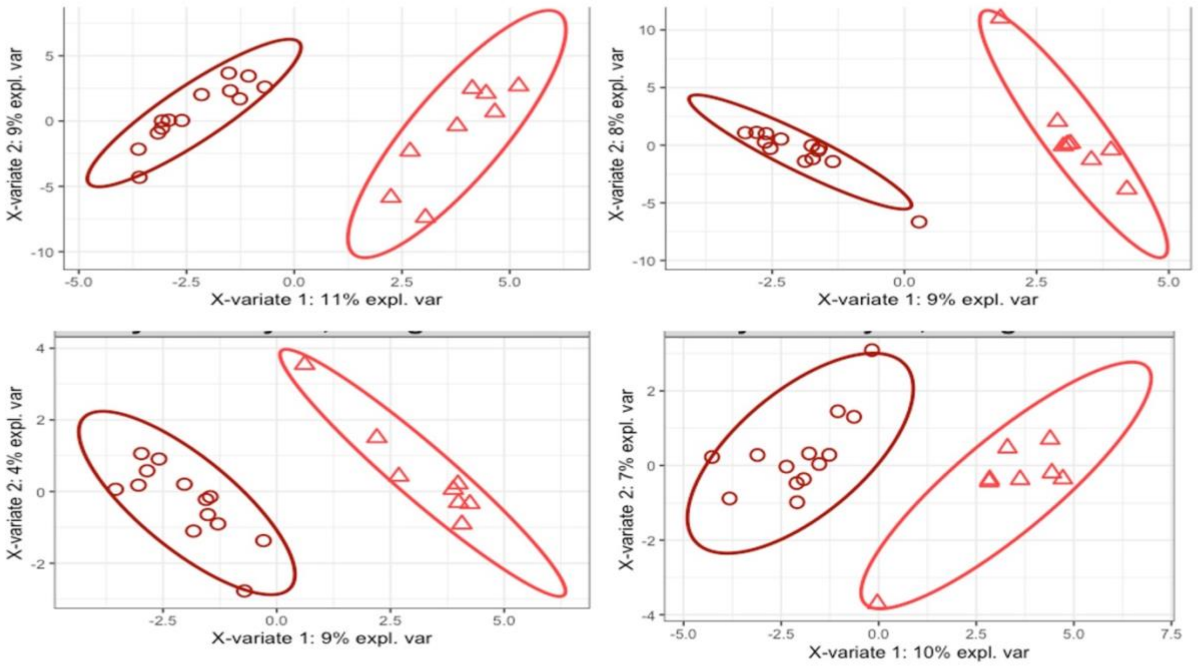

Figure S10: Sparse Partial Least Squares Discriminant analysis of patient randomised to ketogenic feeding on Day 1 (red sphere) and day 10 (red triangle). Clockwise from top right: polar positive, non-polar positive, non-polar negative, polar negative. Error rates are <20% (10%, 12%, 15% and 10% respectively), suggesting plot is result of true variance. N=14 subjects in the ketogenic arm and n=15 subjects in the control arm.

Supplementary References

1. Domingo-Almenara X, Siuzdak G. Metabolomics Data Processing Using XCMS. Methods Mol Biol 2020;**2104**:11-24.
2. Broadhurst D, Goodacre R, Reinke SN, et al. Guidelines and considerations for the use of system suitability and quality control samples in mass spectrometry assays applied in untargeted clinical metabolomic studies. Metabolomics 2018;**14**(6):72.
