# Supplementary material for: A pilot study of alternative substrates in the critically Ill subject using a ketogenic feed
Source: Nat Commun. 2023 Dec 15;14:8345. doi: 10.1038/s41467-023-42659-8 (PMC10724188; doi:10.1038/s41467-023-42659-8)
Supplement: Supplementary file 3 — Reporting Summary [file 41467_2023_42659_MOESM3_ESM.pdf]

nature portfolio

Corresponding author(s): Zudin Amilka Puthucheary

Last updated by author(s): Sep 29, 2023

Reporting Summary

Nature Portfolio wishes to improve the reproducibility of the work that we publish. This form provides structure for consistency and transparency in reporting. For further information on Nature Portfolio policies, see our Editorial Policies and the Editorial Policy Checklist.

Statistics

For all statistical analyses, confirm that the following items are present in the figure legend, table legend, main text, or Methods section.

n/a	Confirmed	
☐	☒	The exact sample size (n) for each experimental group/condition, given as a discrete number and unit of measurement
☐	☒	A statement on whether measurements were taken from distinct samples or whether the same sample was measured repeatedly
☐	☒	The statistical test(s) used AND whether they are one- or two-sided<br>*Only common tests should be described solely by name; describe more complex techniques in the Methods section.*
☐	☒	A description of all covariates tested
☐	☒	A description of any assumptions or corrections, such as tests of normality and adjustment for multiple comparisons
☐	☒	A full description of the statistical parameters including central tendency (e.g. means) or other basic estimates (e.g. regression coefficient) AND variation (e.g. standard deviation) or associated estimates of uncertainty (e.g. confidence intervals)
☐	☒	For null hypothesis testing, the test statistic (e.g. F, t, r) with confidence intervals, effect sizes, degrees of freedom and P value noted<br>*Give P values as exact values whenever suitable.*
☒	☐	For Bayesian analysis, information on the choice of priors and Markov chain Monte Carlo settings
☒	☐	For hierarchical and complex designs, identification of the appropriate level for tests and full reporting of outcomes
☒	☐	Estimates of effect sizes (e.g. Cohen's d, Pearson's r), indicating how they were calculated

Our web collection on statistics for biologists contains articles on many of the points above.

Software and code

Policy information about availability of computer code

Data collection	no software was used
Data analysis	Metaboanalyst 5.0 (www.metaboanalyst.ca/MetaboAnalyst/home.xhtml); Graphpad Prism 8.0 (www.graphpad.com); R version 4.0.3 (https://cran.r-project.org/) and R Studio (version 1.3.1093)

For manuscripts utilizing custom algorithms or software that are central to the research but not yet described in published literature, software must be made available to editors and reviewers. We strongly encourage code deposition in a community repository (e.g. GitHub). See the Nature Portfolio guidelines for submitting code & software for further information.

Data

Policy information about availability of data

All manuscripts must include a data availability statement. This statement should provide the following information, where applicable:

- Accession codes, unique identifiers, or web links for publicly available datasets
- A description of any restrictions on data availability
- For clinical datasets or third party data, please ensure that the statement adheres to our policy

Metabolomic data that support the findings of this study are available for research purposes on reasonable request from the corresponding author [ZAP] who will respond within 14 days. The data are not publicly available since this was not included in research participant consent. No identifiable data will be shared.

Human research participants

Policy information about studies involving human research participants and Sex and Gender in Research.

Reporting on sex and gender	Sex was reported as determined by the next of kin (while participants were unconscious). Findings apply universally, regardless of sex or gender. This was anticipated to be the case, and neither sex nor gender were considered in the study design. Disaggregated sex and gender data were not collected and consent has not been obtained for sharing of individual level data. No sex and gender based analyses were performed, as there was not anticipated to be any form of interaction.
Population characteristics	age 52.0 (45.5-58.5); admission diagnoses were: sepsis (6.9%) Cardiogenic Shock (3.5%), Trauma (31.0%) Res
Recruitment	Patients were recruited by research nurses on the Intensive Care Units,for inclusion on weekdays, being eligible for enrolment up to 48 hours after ICU admission. Prospective informed assent was by nominated personal consultee (in person or by telephone) or professional consultee. Retrospective participant consent was obtained on return of each participant's mental capacity. Permission to use participants' data if capacity did not return or if they did not survive, was included in the assent process.
Ethics oversight	The study received ethics committee approval (National Research Ethics Service Committee Wales 5 – Bangor; REC reference 19/WA/0209; IRAS project ID 266031)

Note that full information on the approval of the study protocol must also be provided in the manuscript.

Field-specific reporting

Please select the one below that is the best fit for your research. If you are not sure, read the appropriate sections before making your selection.

☒ Life sciences ☐ Behavioural & social sciences ☐ Ecological, evolutionary & environmental sciences

For a reference copy of the document with all sections, see nature.com/documents/nr-reporting-summary-flat.pdf

Life sciences study design

All studies must disclose on these points even when the disclosure is negative.

Sample size	Sample sizes of 12 per arm have been recommended where previous data on which to base a power calculation are lacking. We aimed to recruit at least 37 patients to allow for a possible high drop-out rate from early death and early recovery, and for protocol violations (common in many critical care trials), and to thus leave12 patients per arm. From our previous multi-centre trials and observational studies in critically ill patients with multi-organ failure , drop out levels can be of this magnitude due to early death post-recruitment, unexpected discharge from ICU, extubation earlier than expected, protocol violations and clinical factors (such as a patient not meeting nutritional requirements) leading to the managing clinician transferring them to standard feed.
Data exclusions	No data were excluded
Replication	N/A - clinical trial so no replication
Randomization	Randomisation was stratified for recruitment site (1:1 basis) and occurred once assent was obtained and participants enrolled by research staff. An independent remote electronic web-based random allocation service to generated an unpredictable treatment arm allocation, and concealed that outcome from research staff until assignment occurred.
Blinding	Whilst patients were blinded (by virtue of illness), knowledge of the feeding intervention they were receiving would not influence outcome. Where possible individuals collecting outcome assessments were blinded in this feasibility study, but not those preparing and administering feed due COVID-19-related staffing limitations. DB (who assessed all ultrasound scans) was masked to allocation until data analysis was complete (see Supplementary Information).

Reporting for specific materials, systems and methods

We require information from authors about some types of materials, experimental systems and methods used in many studies. Here, indicate whether each material, system or method listed is relevant to your study. If you are not sure if a list item applies to your research, read the appropriate section before selecting a response.

Materials & experimental systems

n/a	Involved in the study
☒	☐ Antibodies
☒	☐ Eukaryotic cell lines
☒	☐ Palaeontology and archaeology
☒	☐ Animals and other organisms
☐	☒ Clinical data
☒	☐ Dual use research of concern

Methods

n/a	Involved in the study
☒	☐ ChIP-seq
☒	☐ Flow cytometry
☒	☐ MRI-based neuroimaging

Clinical data

Policy information about clinical studies

All manuscripts should comply with the ICMJEguidelines for publication of clinical research and a completedCONSORT checklist must be included with all submissions.

Clinical trial registration: ClinicalTrials.gov, NCT04101071

Study protocol: Full trial protocol was not made available publicly, but is available on request.

Data collection: Data collection was performed on the ICU and/or hospital wards (daily physiology, blood and urine sampling), physical performance measures and muscle ultrasound (at ICU discharge and hospital discharge) and by telephone for follow up (longer term outcomes)

Outcomes: The primary endpoints were patient recruitment and consent rates during screening (with retention rates); the ease of reconstitution and administration of the novel ketogenic enteral feed by ICU and research staff (determined via questionnaire; safety (reports of adverse events [AEs] and serious adverse events [SAEs]); parameters of enteral feed absorption and blood chemistry (including glucose levels and achievement of ketosis) post-recruitment; and plasma concentrations of beta hydroxybutyrate and acetoacetate, glucose, lactate, pyruvate and medium-chain fatty acids from blood samples at timepoints during the 10-day study period.

Secondary endpoints included arterial blood gas parameters, ultrasound-determined rectus femoris cross-sectional area (as a marker of muscle loss); clinical, physiological and nutritional data; non-invasive metabolic data via indirect calorimetry; measures of physical function and quality of life; employment status and health care resource usage; and urinary concentrations of beta-hydroxybutyrate and total nitrogen, and plasma metabolomics at timepoints during the 10-day study period.
